# Two Novel Fluorescence Probes Based on Caffeic Acid Derivative for Phosphate Ions and Their Applications in Biological Samples

**DOI:** 10.3390/ijms252111680

**Published:** 2024-10-30

**Authors:** Xiaowen Zhou, Xiaoqin Yang, Xiaoping Rao, Yingjun Zhang, Ping Zhao, Qian Jiang

**Affiliations:** 1 Key Laboratory of State Forestry and Grassland Administration on Highly-Efficient Utilization of Forestry Biomass Resources in Southwest China, Southwest Forestry University, Kunming 650224, China; zhouxw0504@outlook.com (X.Z.); yangxiaoqin@swfu.edu.cn (X.Y.); hypzhao@swfu.edu.cn (P.Z.); 2 College of Chemical Engineering, Huaqiao University, Xiamen 361021, China; raoxp@hqu.edu.cn; 3 Laboratory of Phytochemistry and Plant Resources in West China, Kunming Institute of Botany, Chinese Academy of Sciences, Kunming 650204, China; zhangyj@mail.kib.ac.cn

**Keywords:** fluorescent probe, caffeic acid derivative, highly selective, phosphates, cell imaging

## Abstract

Phosphate is widely used in industry and agriculture fields. However, excess accumulation of PO_4_^3−^ causes several adverse effects on the human body and ecological environment. Consequently, it is important to develop a simple method for the detection of PO_4_^3−^ concentration in the ecological environment and in vivo. Herein, two caffeic acid derivative-based fluorescence probes (**BAM-HM** and **BAM-HH**) were developed for the detection of phosphate. The **BAM-HM** probe could detect phosphate via fluorescence enhancement at 500 nm, with the detection limit being 0.612 µM. Meanwhile, the **BAM-HH** probe showed a significant turn-on signal at 450 nm after the addition of phosphate, and the detection limit was calculated to be 0.318 µM. The sensing mechanism was determined by ^1^H NMR and MS. Furthermore, the two probes (**BAM-HM** and **BAM-HH**) were applied for PO_4_^3−^detection in living cells and water samples.

## 1. Introduction

Because of its unique chemical properties, phosphate (PO_4_^3−^) plays an important role in many industrial and agricultural fields, such as chemical reagents, fertilizers, foods, pharmaceuticals, and animal feeds [1,2,3]. In the food industry, phosphate ions are often applied in the production of food additives. As a necessary nutrient element for plant growth, phosphorus is transformed into plant auxin after entering the plant [4,5]. Currently, due to the wide application of phosphate, a large amount of phosphate is released into the aquatic environment and ultimately enters the human body through the food chain [6,7,8]. Regarding human health, studies have shown that excessive phosphate intake may interfere with the absorption of calcium, lead to an imbalance of calcium and phosphorus, and increase the risk of bone disease [9,10]. In addition, excessive intake of phosphate ions may also burden the kidneys and aggravate pre-existing kidney disease [11]. Therefore, it is essential to determine the changes in phosphate content in biological processes.

To date, several traditional analytical techniques have been developed for the detection of PO_4_^3−^, such as liquid or gas chromatography–mass spectrometry, surface-enhanced Raman spectroscopy, high-performance liquid chromatography, and electrochemistry [12,13,14,15]. Compared with these traditional detection methods, the fluorescence analysis method can be used as an effective analytical tool because of its advantages, such as high sensitivity and selectivity, convenience, real-time detection, and low cost [16,17,18,19]. To date, a large number of fluorescence probes with excellent fluorescent properties for the detection of biomolecules have been designed [20,21,22,23]. Among these reported specific fluorescence probes, few fluorescence probes for the detection of phosphate have been reported [24,25,26,27,28]. Therefore, it is still a challenge to design PO_4_^3−^ fluorescence probes with high sensitivity and selectivity that can be applied for detection in living cells.

Due to its outstanding pharmacological activities, including antidepressant, antioxidant, and anticancer effects, caffeic acid (CA) is regarded as a bioactive molecule in plants [29,30,31,32] and is often employed in the design of drug molecules. Nevertheless, the structure of CA contains one phenyl group and two hydroxyl groups, which is beneficial for the enhancement of fluorescence intensity. Currently, reports of fluorescent probes based on CA are still rare [33]. As a result, developing a unique fluorescent probe based on CA is quite desirable.

Herein, three CA derivatives (**BAM-HM**, **BAM-MM**, and **BAM-HH**) were successfully synthesized. **BAM-HM** and **BAM-HH** could be used as PO_4_^3−^ probes. These two PO_4_^3−^ probes possess some advantages, such as high selectivity, a low detection limit (**BAM-HM**, 0.612 µM; **BAM-HH**, 0.318 µM), and real-time detection. Furthermore, for practical applications, the **BAM-HM** and **BAM-HH** probes are capable of determining the PO_4_^3−^ concentration in living cells and real water.

## 2. Results and Discussion

### 2.1. Synthesis

In this work, three caffeic acid derivatives were designed and synthesized through a simple synthetic route (Scheme 1). All the final products were purified and characterized by ^1^H NMR, ^13^C NMR, and HRMS, which confirmed their molecular structures (Figures S1–S9).

### 2.2. Photophysical Properties of **BAM-HM**, **BAM-MM**, and **BAM-HH**

**Figure 1:**
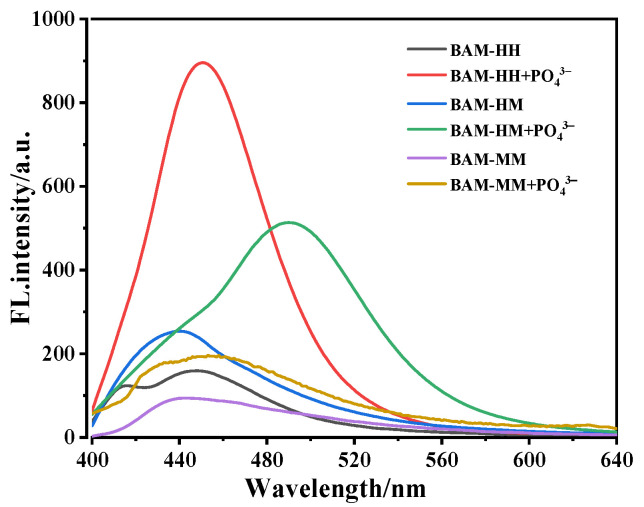
Fluorescence spectra of three compounds, **BAM-HM**, **BAM-MM**, and **BAM-HH**, in the presence of PO_4_^3−^ in DMF/H_2_O (pH = 7.0, 50% DMF).

Initially, we studied the photophysical properties of **BAM-HM**, **BAM-MM**, and **BAM-HH**. As shown in Figure 1, the three compounds all displayed weak fluorescence, and the fluorescence quantum yields (φ) of **BAM-HM**, **BAM-MM**, and **BAM-HH** were 0.0231, 0.014, and 0.016. However, in the solution of **BAM-HM**, the addition of PO_4_^3−^ triggered a new emission peak at 500 nm, accompanied by green fluorescence (φ = 0.361). In addition, after the addition of PO_4_^3−^, the fluorescence emission peak of **BAM-HH** at 450 nm appeared, and obvious blue fluorescence was observed (φ = 0.458). Finally, no significant fluorescence enhancement appeared in the solution of **BAM-MM** + PO_4_^3−^ (φ = 0.017). Figure S10 shows the UV–vis spectral changes upon the addition of PO_4_^3−^. **BAM-HM**, **BAM-MM,** and **BAM-HH** showed obvious absorption maxima at around 320 nm, 320 nm, and 300 nm, respectively. Then, upon the addition of PO_4_^3−^ to the **BAM-MM** compound, no obvious absorption band change appeared in the UV–vis spectra. Surprisingly, upon the addition of PO_4_^3−^ to the solutions of **BAM-HM** and **BAM-HH**, both absorption maxima at 320 nm and 300 nm decreased significantly, and two new absorption peaks appeared at 370 nm and 354 nm, respectively. These results indicated that **BAM-HM** and **BAM-HH** could be employed as fluorescence probes for PO_4_^3−^ detection.

### 2.3. Spectroscopic Response of Probe **BAM-HM** to PO_4_^3−^

**Figure 2:**
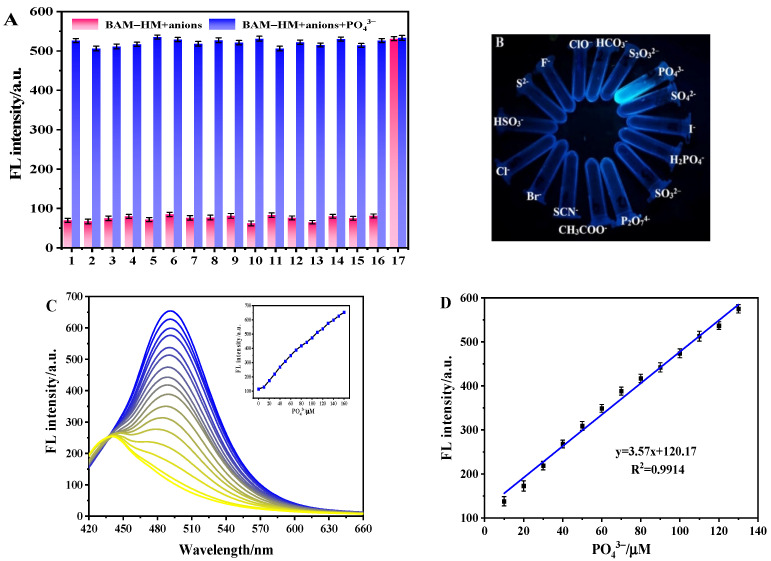
(**A**) Selectivity and interference of the **BAM-HM** probe for anions (1–16: F^−^, Cl^−^, Br^−^, I^−^, HCO_3_^−^, CO_3_^2−^, AcO^−^, SCN^−^, SO_3_^2−^, ClO^−^, HSO_3_^−^, SO_4_^2−^, S^2−^, S_2_O_3_^2−^, HPO_4_^2−^, PO_4_^3−^). (**B**) Fluorescence photos of the **BAM-HM** probe before and after adding different anions. (**C**) Fluorescence spectra of different concentrations of PO_4_^3−^ were added to the solution of the **BAM-HM** probe. (**D**) Linear relationship between fluorescence intensity and PO_4_^3−^ concentration.

First, the selectivity of the **BAM-HM** probe toward various anions and cations was investigated; as shown in Figure 2A and Figure S11, only PO_4_^3−^ led to a significant fluorescence change, and other ions were not found in any variations. Meanwhile, the fluorescence solution of the **BAM-HM** probe changed from colorless to green (Figure 2B). In addition, an obvious fluorescence intensity enhancement at 500 nm was observed after the addition of PO_4_^3−^ to the solution of the **BAM-HM** probe.

We observed that the complex showed a mole fraction with **BAM-HM** of 0.5 for PO_4_^3−^, which meant a 1:1 binding stoichiometry (Figure S12). Then, the sensitivity of the **BAM-HM** probe was studied using a titration experiment. As shown in Figure 2C, as the concentration of PO_4_^3−^ increased from 0 µM to 160 µM, the fluorescence intensity of the **BAM-HM** probe at 500 nm increased gradually. The fluorescence intensity of the **BAM-HM** probe showed a good linear relationship with the PO_4_^3−^ concentration (Figure 2D), and the detection limit of **BAM-HM** was calculated to be 0.612 µM according to the formula LOD = 3σ/K [34,35,36,37].

### 2.4. Spectroscopic Response of Probe **BAM-HH** to PO_4_^3−^

**Figure 3:**
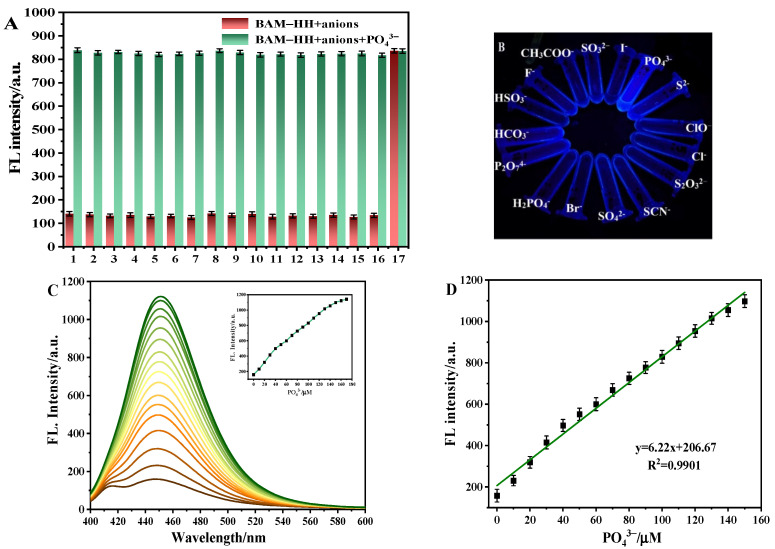
(**A**) Selectivity and interference of the **BAM-HH** probe for anions (1–16: F^−^, Cl^−^, Br^−^, I^−^, HCO_3_^−^, CO_3_^2−^, AcO^−^, SCN^−^, SO_3_^2−^, ClO^−^, HSO_3_^−^, SO_4_^2−^, S^2−^, S_2_O_3_^2−^, HPO_4_^2−^, PO_4_^3−^). (**B**) Fluorescence photos of the **BAM-HH** probe before and after adding different anions. (**C**) Fluorescence spectra of different concentrations of PO_4_^3−^ were added to the solution of the **BAM-HH** probe. (**D**) Linear relationship between fluorescence intensity and PO_4_^3−^ concentration.

The fluorescent spectra responses of the **BAM-HH** probe toward various ions were studied using fluorescent spectra analytes. Only the introduction of PO_4_^3−^ led to an obvious green fluorescence enhancement (Figure 3A and Figure S13). In addition, the addition of other anions and cations did not induce a significant fluorescence change (Figure 3B). The anti-interference experiment indicated that the presence of anions and cations caused a negligible impact on PO_4_^3−^ detection.

Subsequently, the binding stoichiometry of **BAM-HH** exhibited a 1:1 ratio for PO_4_^3−^ sensing (Figure S14). Then, fluorescence titrations for PO_4_^3−^ were performed in a DMF/H_2_O (5/5, *V*/*V*) solution. As shown in Figure 3C, with the addition of PO_4_^3−^ (0–170 µM), the maximum emission peak of the **BAM-HH** probe increased gradually, and the fluorescence intensities of the **BAM-HH** probe were linearly correlated with the PO_4_^3−^ concentrations (Figure 3D). The detection limit of **BAM-HH** was calculated to be 0.318 µM. These results suggested that **BAM-HM** and **BAM-HH** are highly sensitive toward PO_4_^3−^ [38,39,40,41,42] (Table S1).

### 2.5. Sensing Time and Stability Experiments

**Figure 4:**
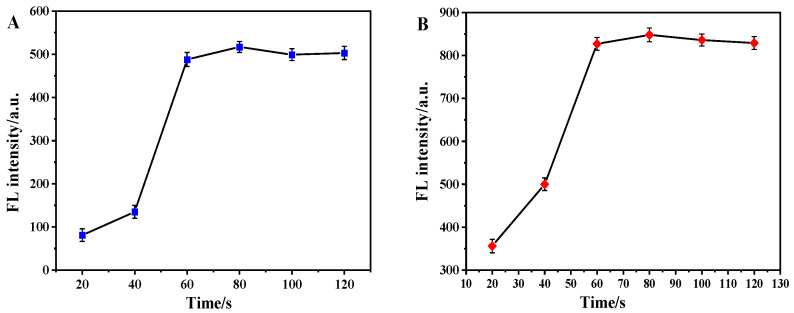
Fluorescence response time for the **BAM-HM** (**A**) and **BAM-HH** (**B**) probes with the addition of PO_4_^3−^.

To further elucidate the response time of the **BAM-HM** and **BAM-HH** probes to detect PO_4_^3−^, the fluorescence intensity of the **BAM-HM** and **BAM-HH** probes at different sensing times was recorded after adding PO_4_^3−^. With an increase in response time, the fluorescence intensity increased continuously. The fluorescence intensity of **BAM-HM** + PO_4_^3−^ at 500 nm reached its maximum value in 80 s and stayed almost unchanged at subsequent times (Figure 4A). Meanwhile, after the addition of PO_4_^3−^, the **BAM-HH** probe also showed a shorter response time toward PO_4_^3−^ within 60 s (Figure 4B), which indicated that the **BAM-HM** and **BAM-HH** probe solutions could instantaneously determine PO_4_^3−^.

Next, we studied the influence of the variation in pH values on the fluorescence performance of the **BAM-HM** and **BAM-HH** probes toward PO_4_^3−^. The fluorescent intensities of **BAM-HM** + PO_4_^3−^ (Figure S15A) and **BAM-HH** + PO_4_^3−^ (Figure S15B) increased in the range of pH 1–3 and showed good stability at diverse pH levels (from 4 to 11). These results suggest that **BAM-HM** and **BAM-HH** have the capability to identify PO_4_^3−^ at a wide range of pH levels.

### 2.6. Mechanism Studies

**Figure 5:**
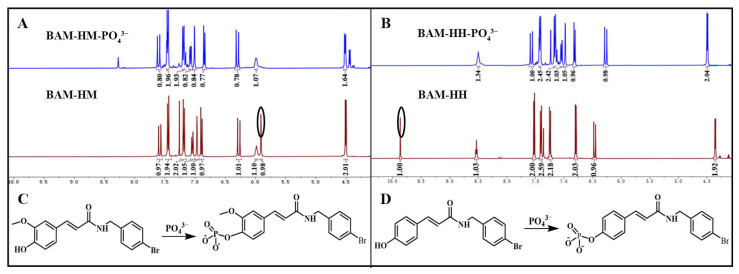
^1^H NMR spectra of the **BAM-HM** (**A**) and **BAM-HH** (**C**) probes before and after adding PO_4_^3−^. The binding mechanism of the **BAM-HM** (**B**) and **BAM-HH** (**D**) probes.

To further investigate the sensing mechanisms of the two probes to PO_4_^3−^, ^1^H NMR and MS analyses in the presence of PO_4_^3−^ were performed. As shown in Figure 5A, the hydroxyl group (Ha) peak in the **BAM-HM** probe appeared at 5.91 ppm and disappeared upon complexation with PO_4_^3−^. Then, it was found that the hydroxyl group (Hb) of the **BAM-HH** probe at 9.87 ppm disappeared after PO_4_^3−^, which indicated that PO_4_^3−^ caused the deprotonation of the hydroxyl group (Figure 5C). The proposed mechanism is shown in Figure 5B, D. In addition, in the MS spectra of **BAM-HM** + PO_4_^3−^ and **BAM-HH** + PO_4_^3−^, a new peak at 439.2011 corresponding to **BAM-HM** + PO_4_^3−^ was observed (Figure S16A). According to Figure S16B, the peak at *m*/*z* 409.0913 supports the structure of **BAM-HH** + PO_4_^3−^. The above results further support the proposed mechanism.

**Figure 6:**
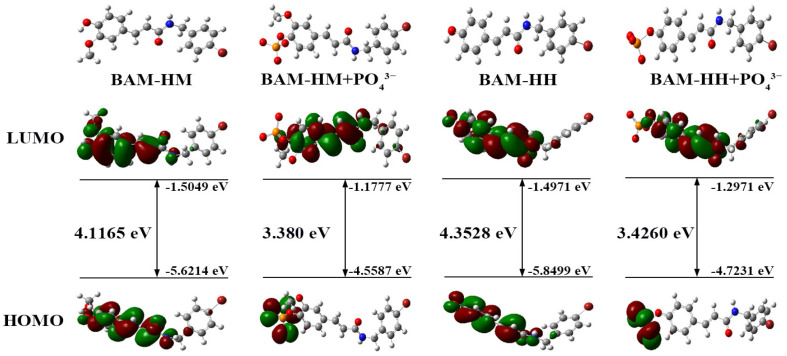
Frontier molecular orbital profiles of **BAM-HM**, **BAM-HH**, **BAM-HM** + PO_4_^3−^, and **BAM-HH** + PO_4_^3−^.

The results of **BAM-HM**, **BAM-HM** + PO_4_^3−^, **BAM-HH,** and **BAM-HH** + PO_4_^3−^ are shown in Figure 6. In the **BAM-HM** probe, the HOMO and LUMO orbitals are mainly distributed on feruloyl, with an energy band gap of 4.1165 eV. The HOMO and LUMO orbitals of the **BAM-HH** probe are mainly distributed on 4-hydroxycinnamoyl, with the energy level calculated at 4.3528 eV. A small amount of electron transfer in **BAM-HM** and **BAM-HH** led to weak fluorescence. From the calculation results, it could be seen that the LUMO of **BAM-HM** + PO_4_^3−^ was located in feruloyl, and the HOMO was distributed at the center of the PO_4_^3−^ ligand. In the **BAM-HH** + PO_4_^3−^, the electron clouds in the LUMO were distributed on the 4-hydroxycinnamoyl, and the HOMO was located at the PO_4_^3−^ center of **BAM-HH** + PO_4_^3−^. The energy gaps of **BAM-HM** + PO_4_^3−^ and **BAM-HH** + PO_4_^3−^ were 3.380 eV and 3.460 eV, respectively. The energy gaps of **BAM-HM** + PO_4_^3−^ and **BAM-HH** + PO_4_^3−^ were smaller than those of **BAM-HM** and **BAM-HH**, suggesting that **BAM-HM** and **BAM-HH** tend to bind PO_4_^3−^, and the combined complex is more stable. The energy gap of **BAM-HM** + PO_4_^3−^ was smaller than that of **BAM-HH** + PO_4_^3−^, indicating that the intramolecular charge transfer (ICT) process of **BAM-HH** + PO_4_^3−^ was weakened compared with that of **BAM-HM** + PO_4_^3−^. The above results were consistent with the experimental results.

### 2.7. Application in Real Water Samples

Next, the practical application of the two probes was investigated, and we estimated the PO_4_^3−^ concentration in water samples via two probes. Different concentrations of PO_4_^3−^ were added to water samples. First, the fluorescence intensity of **BAM-HM** and **BAM-HH** mixed with different PO_4_^3−^ concentrations (2, 5, 10, and 15 µM) was recorded. The corresponding recoveries are shown in Tables S2 and S3. The recovery rate for PO_4_^3−^ranged from 96 to 105%, indicating that **BAM-HM** and **BAM-HH** have the ability to evaluate the level of PO_4_^3−^ in real water samples.

### 2.8. Cell Imaging

Prior to the fluorescence imaging experiment, the toxicity of the **BAM-HM** and **BAM-HH** probes in HeLa cells was studied using the CCK8-assay. HeLa cells were treated with different concentrations (0–200 µM) of the two compounds for 24 h. As shown in Figure S17, the cell survival ratios of the two compounds were above 80% at a concentration of 100 µM. These results suggested that **BAM-HM** and **BAM-HH** showed low cytotoxicity and could be used for cell imaging experiments.

**Figure 7:**
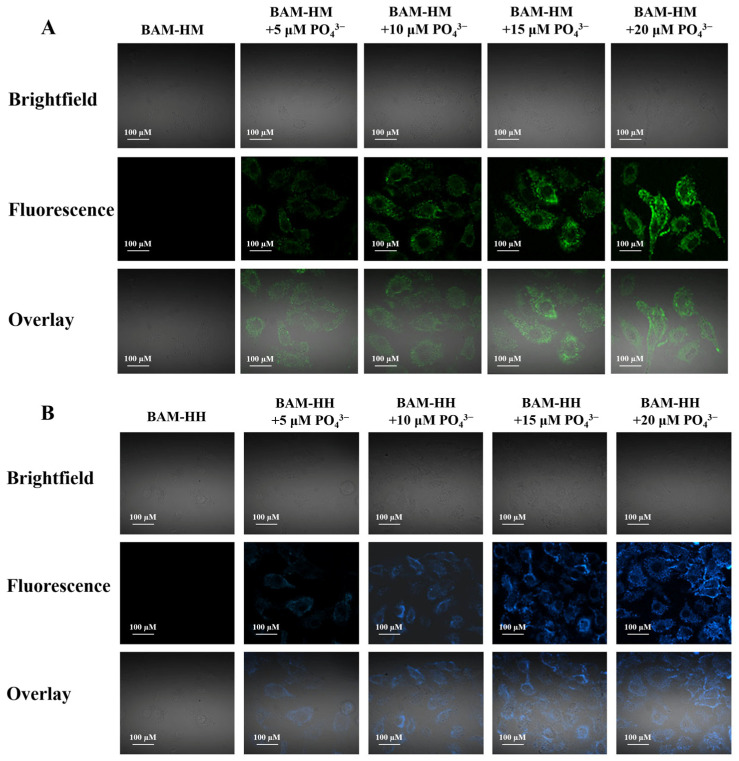
The fluorescence imaging of HeLa cells treated with the **BAM-HM** (**A**) and **BAM-HH** (**B**) probes and then incubated with different concentrations of PO_4_^3−^ (5 µM, 10 µM, 15 µM, and 20 µM).

Then, the capacity of the **BAM-HM** and **BAM-HH** probes was examined in HeLa cells. As displayed in Figure 7A, when the cells were incubated with the **BAM-HM** probe, no obvious fluorescence was observed. Then, the pre-treated cells were treated with different concentrations of PO_4_^3−^ (0, 5 µM, 10 µM, 15 µM, and 20 µM), and a faint green fluorescence was observed; the intracellular green fluorescence was clearly enhanced with an increase in PO_4_^3−^ concentration. In addition, the HeLa cells were incubated with the **BAM-HH** probe, which showed weak fluorescence. The **BAM-HH**-loaded HeLa cells were further treated with different concentrations of PO_4_^3−^ (0, 5 µM, 10 µM, 15 µM, and 20 µM). With an increase in PO_4_^3−^ concentration, an obvious fluorescence enhancement in the blue channel was observed (Figure 7B). The cell experiments demonstrate that the **BAM-HM** and **BAM-HH** probes can be potentially used as a powerful tool for the detection of PO_4_^3−^ in vivo.

## 3. Materials and Methods

### 3.1. General Instrumentations and Reagents

The chemicals and solvents in this study were purchased from chemical suppliers (Titan, shanghai, China) and used as received without further purification. Compounds were characterized by ^1^H and ^13^C NMR spectra (Bruker, Billerica, MA, USA) and ESI-MS (Agilent, Santa Clara, CA, USA). The fluorescence spectrum of the probe was recorded using a fluorescence spectrometer (Hitachi, Kyoto, Japan). The pH measurements were recorded by a pH measuring apparatus (Ohaus, NJ, USA). The purity of the compounds was recorded using an LCMS spectrometer (Agilent, CA, USA). The fluorescence cell imaging was performed using a confocal laser scanning microscope (Leica, Wetzlar, Germany).

### 3.2. General Synthetic Procedure for **BAM-HM**, **BAM-MM**, and **BAM-HH**

**BAM-HM**: 4-hydroxy-3-methoxycinnamic acid (138.52 mg, 0.7 mmol), 1-Ethyl-3-(3-dimethylaminopropyl) carbodiimide (EDCl, 134.90 mg, 0.7 mmol) and 1-Hydroxybenzotriazole (HOBT, 95.71 mg, 0.7 mmol) were mixed with 20 mL of DMF. After 20 min, 4-bromobenzylamine (259.81 mg, 1.4 mmol) was added and stirred for another 20 min at room temperature. Then N, N-Diisopropylethylamine (180.36 mg, 1.4 mmol) was added to the aforesaid solution and stirred for 24 h. After the reaction was completed, the mixture was extracted with ethyl acetate, and the residue was obtained after the solvent was removed. Then, the residue was purified using silica gel column chromatography (PE/EA = 5/1) to yield the product, which was a pale yellow solid (93.22 mg, 68.6%). ^1^H NMR (400 MHz, CDCl_3_-*d*_6_) δ 7.58 (d, *J* = 15.5 Hz, 1H), 7.49–7.39 (m, 2H), 7.25–7.15 (m, 2H), 7.04 (dd, *J* = 8.2, 2.0 Hz, 1H), 6.97 (d, *J* = 1.9 Hz, 1H), 6.89 (d, *J* = 8.2 Hz, 1H), 6.28 (d, *J* = 15.6 Hz, 1H), 5.98 (s, 1H), 5.91 (s, 1H), 4.51 (d, *J* = 5.9 Hz, 2H), 3.89 (s, 3H). ^13^C NMR (126 MHz, DMSO-*d*_6_) δ 165.48, 148.36, 147.81, 139.51, 139.14, 131.15, 129.51, 126.29, 121.59, 119.74, 118.57, 115.65, 110.82, 55.51, 41.61. ESI-MS *m*/*z*: [M + H]^+^ calculated for C_17_H_16_BrNO_3_ 362.0215, found 362.0589.

**BAM-MM**: **BAM-MM** was prepared using 3,4-dimethoxycinnamic acid (124 mg, 0.6 mmol) instead of 4-hydroxy-3-methoxycinnamic acid, according to a similar procedure for **BAM-HM**. The product was a yellow solid (164.71 mg, 73.2%). ^1^H NMR (400 MHz, CDCl_3_-*d*_6_) δ 7.60 (d, *J* = 15.5 Hz, 1H), 7.45 (dd, *J* = 8.5, 2.6 Hz, 2H), 7.21–7.17 (m, 2H), 7.07 (dd, *J* = 8.3, 2.1 Hz, 1H), 7.01 (d, *J* = 2.0 Hz, 1H), 6.85 (d, *J* = 8.3 Hz, 1H), 6.30 (d, *J* = 15.5 Hz, 1H), 5.99 (s, 1H), 4.51 (d, *J* = 5.8 Hz, 2H), 3.89 (d, *J* = 5.9 Hz, 6H). ^13^C NMR (126 MHz, DMSO-*d*_6_) δ 165.33, 150.14, 148.88, 139.16, 139.08, 131.15, 129.52, 127.60, 121.40, 119.76, 119.58, 111.73, 110.01, 55.53, 55.40, 41.63. ESI-MS *m*/*z*: [M + H]^+^ calculated for C_18_H_18_BrNO_3_ 376.2604, found 376.2741

**BAM-HH**: **BAM-HH** was prepared using 4-hydroxycinnamic acid (98.43 mg, 0.6 mmol) instead of 4-hydroxy-3-methoxycinnamic acid, according to a similar procedure for **BAM-HM**. The product was a yellow solid (139.42 mg, 70.2%). ^1^H NMR (500 MHz, DMSO-*d*_6_) δ 9.87 (s, 1H), 8.53 (t, *J* = 6.1 Hz, 1H), 7.56–7.49 (m, 2H), 7.44–7.38 (m, 3H), 7.29–7.20 (m, 2H), 6.83–6.76 (m, 2H), 6.46 (d, *J* = 15.7 Hz, 1H), 4.35 (d, *J* = 6.0 Hz, 2H). ^13^C NMR (126 MHz, DMSO-*d*_6_) δ 165.95, 159.39, 139.70, 139.62, 131.63, 130.01, 129.74, 126.28, 120.22, 118.74, 116.22, 42.08. ESI-MS *m*/*z*: [M + H]^+^ calculated for C_16_H_14_BrNO_2_ 332.9208, found 332.9353.

### 3.3. General Protocol Photophysical Properties Experiments

The stock solutions of the **BAM-HM** and **BAM-HH** probes (1 mM) were prepared in a DMF solution (*V*_DMF_/*V*_H2O_ = 1/1, pH = 7), and the test solutions were diluted to a concentration of 10 μM. In the pH and time response experiments, the concentrations of the two probes were maintained at 10 μM, and the test system with various pH levels was adjusted with HCl and NaOH solutions. In the sensitivity experiments, different concentrations of PO_4_^3−^ were added to the solutions of the probes (10 μM). Furthermore, in the selectivity experiments, different anions (I^−^, F^−^, Br^−^, Cl^−^, AcO^−^, HCO_3_^−^, CO_3_^2−^, SCN^−^, SO_3_^2−^, HSO_3_^−^, ClO^−^, SO_4_^2−^, S_2_O_3_^2−^, S^2−^, PO_4_^3−^, and HPO_4_^2−^) and cations (Cr^3+^, Co^2+^, Cd^2+^, Cu^2+^, Ba^2+^, Ni^2+^, Ag^+^, Al^3+^, Zn^2+^, Hg^2+^, Na^+^, Mg^2+^, Mn^2+^, Fe^2+^, and Fe^3+^) were dissolved in deionized water, and the concentration of anions and cations was maintained at 100 μM. All fluorescence measurements were investigated in (*V*_DMF_/*V*_H2O_ = 1/1, pH = 7) at room temperature, the optimal excitation wavelength was set to 350 nm, and the excitation and emission slit widths were set to 10 nm and 10 nm.

### 3.4. Cytotoxicity and Cell Imaging Experiments

The cytotoxicity of the **BAM-HM** and **BAM-HH** probes was estimated with the CCK8-assay [43,44,45,46]. HeLa cells were cultured in an RPMI-1640 medium containing 10% FBS and 1% antibiotics. Then, treated HeLa cells were further incubated with the two different probe solutions (3.13, 6.25, 12.5, 25, 50, 100, and 200 μM) for 24 h. Afterward, each well of plates was further injected with the CCK-8 solution. Four hours later, according to the optical density of the experimental group, the cell viability was determined.

HeLa cells were cultured in an RPMI-1640 medium containing a 10 μM probe solution for 1 h. For cell fluorescence imaging, the probe-pretreated cells were then treated with 5, 10 μM, 15 μM, and 20 μM PO_4_^3−^ for 30 min, respectively. After washing three times with HEPES (2-[4-(2-hydroxyethyl)piperazin-1-yl]ethanesulfonic acid) buffer, the HeLa cells were utilized for cell imaging. Finally, fluorescent photographs of the cells were acquired using a confocal fluorescence microscope.

## 4. Conclusions

In conclusion, we successfully prepared two fluorescence probes (**BAM-HM** and **BAM-HH**) based on a caffeic acid derivative for the detection of PO_4_^3−^. In particular, the two probes can detect PO_4_^3−^ with an obvious fluorescence enhancement in the green channel (**BAM-HM**) and blue channel (**BAM-HH**), respectively. The two probes have multiple advantages, such as good sensitivity (**BAM-HM**, 0.612 µM; **BAM-HH**, 0.318 µM) and selectivity, a wide range of pH applications (3–11), short reaction times (**BAM-HM**, 80 s; **BAM-HH**, 60 s), and low cytotoxicity. The quantitative determination of PO_4_^3−^ in real water samples was investigated. Additionally, the applications of two probes in cell imaging PO_4_^3−^ were demonstrated. Therefore, this study provides an efficient analytical method for the detection and monitoring of PO_4_^3−^ in ecological environments and in vivo.

## Figures and Tables

**Scheme 1:**
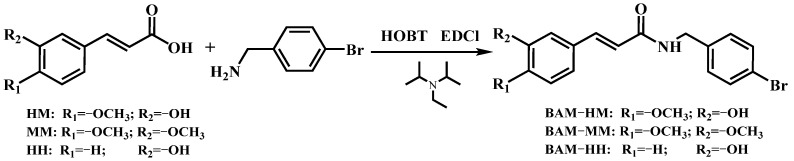
Synthesis route of **BAM-HM**, **BAM-MM** and **BAM-HH**.

## Data Availability

Supplementary Materials: Available: ^1^H and ^13^C NMR spectra and HRMS spectra of **BAM-HM**, **BAM-HH,** and **BAM-MM**.

## Supplementary Materials

The following supporting information can be downloaded at: https://www.mdpi.com/article/10.3390/ijms252111680/s1.
