# Supplementary material for: Two Novel Fluorescence Probes Based on Caffeic Acid Derivative for Phosphate Ions and Their Applications in Biological Samples
Source: Int J Mol Sci. 2024 Oct 30;25(21):11680. doi: 10.3390/ijms252111680 (PMC11546634; doi:10.3390/ijms252111680)
Supplement: Supplementary file 1 [file ijms-25-11680-s001.zip › ijms-3249942-supplementary.pdf]

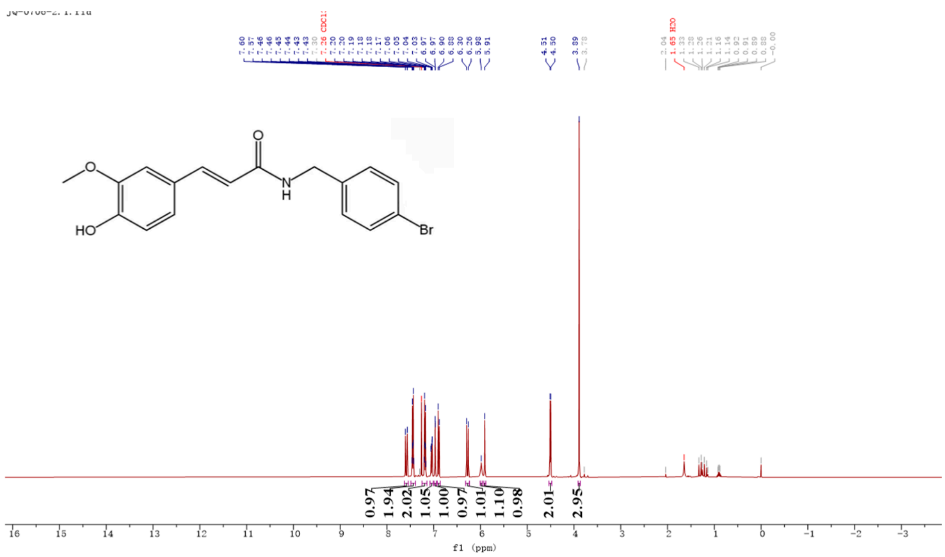

Figure S1 ^{1}H NMR spectrum of compound **BAM-HM** in DMSO.

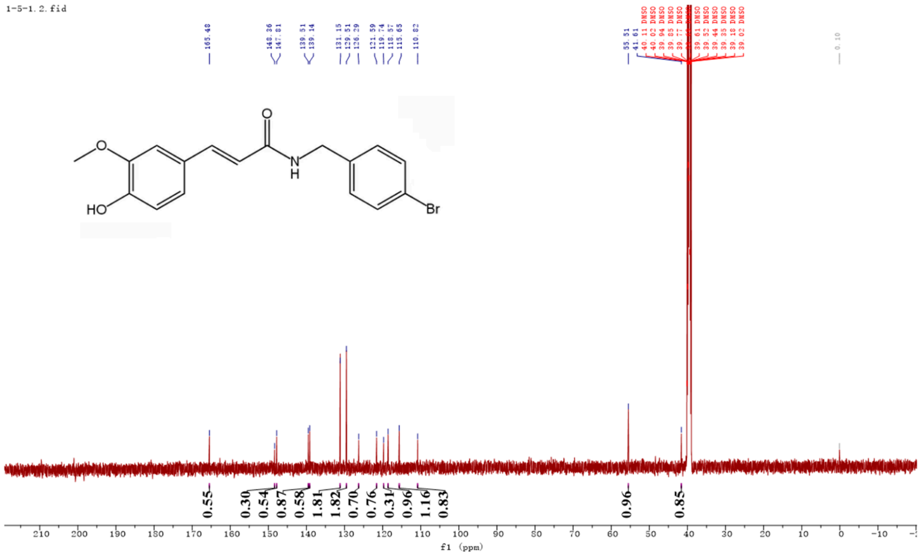

Figure S2 ^{13}C NMR spectrum of compound **BAM-HM** in DMSO.

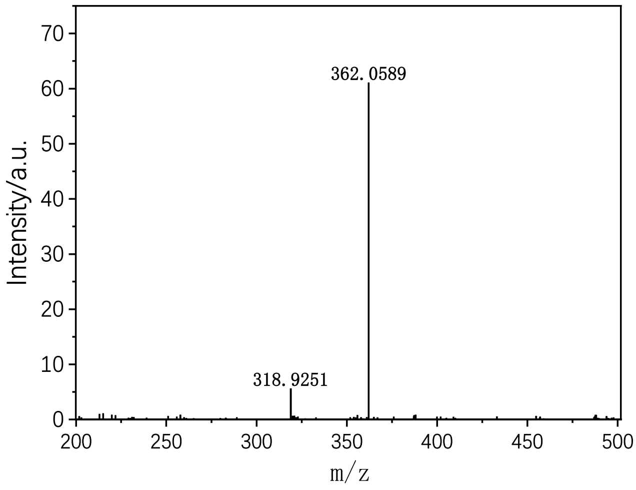

Figure S3 ESI-HRMS spectrum of compound **BAM-HM.**

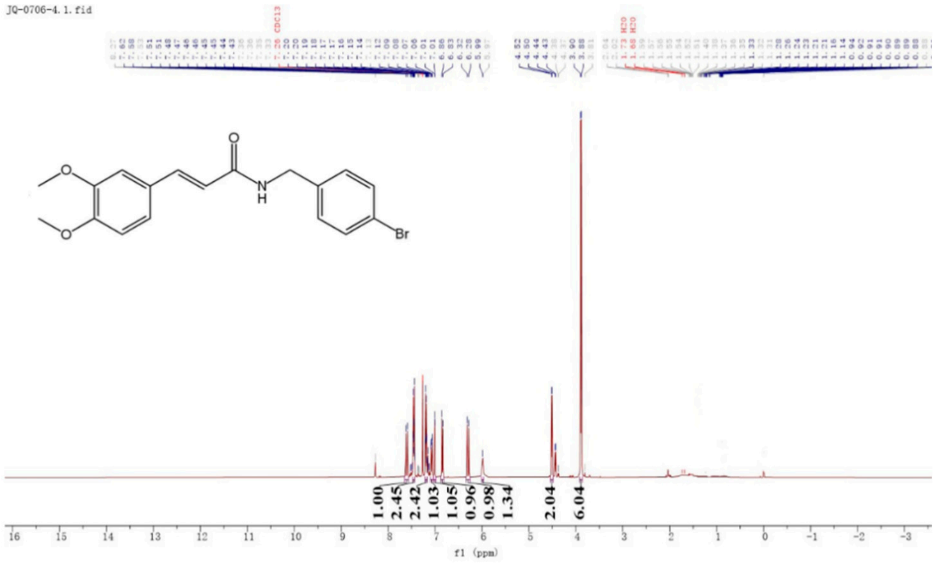

Figure S4 ^{1}H NMR spectrum of compound **BAM-MM** in DMSO.

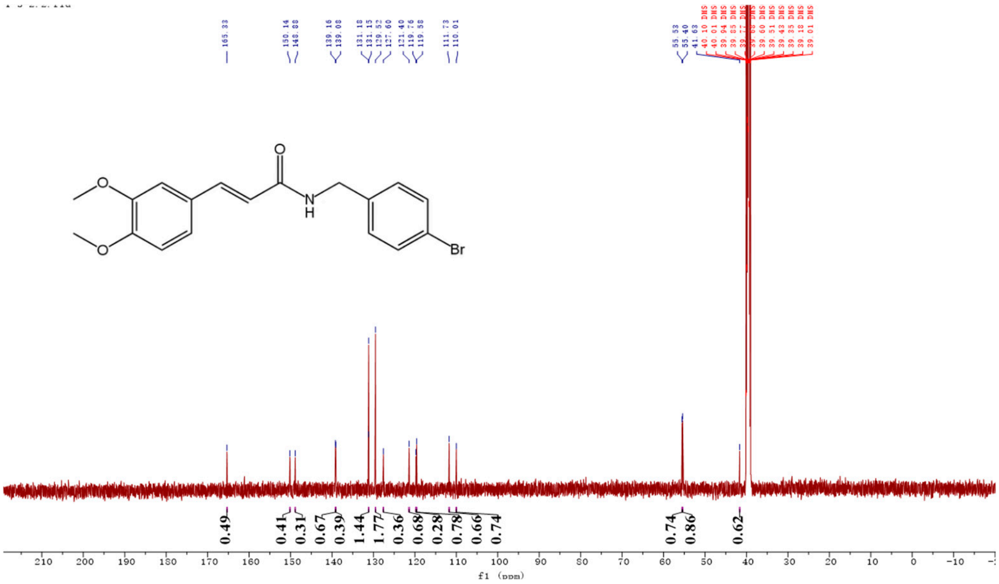

Figure S5 ^{13}C NMR spectrum of compound **BAM-MM** in DMSO.

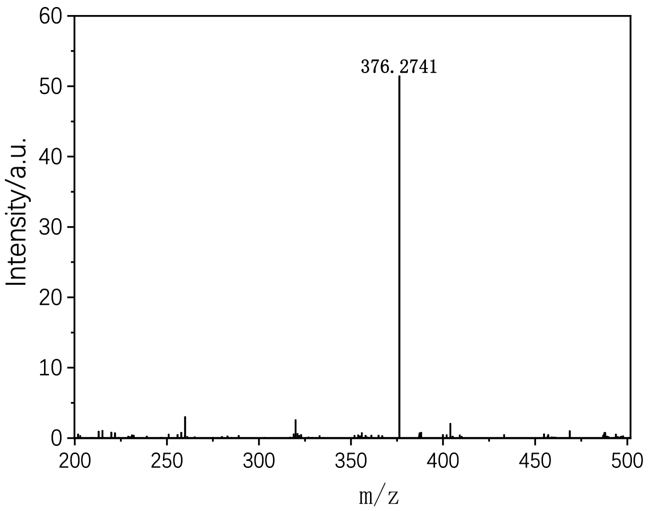

Figure S6 ESI-HRMS spectrum of compound **BAM-MM.**

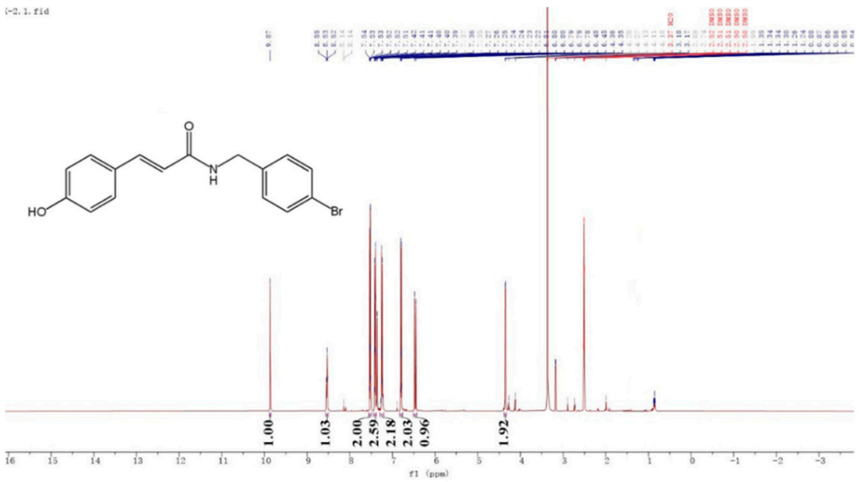

Figure S7 ^{1}H NMR spectrum of compound **BAM-HH** in DMSO.

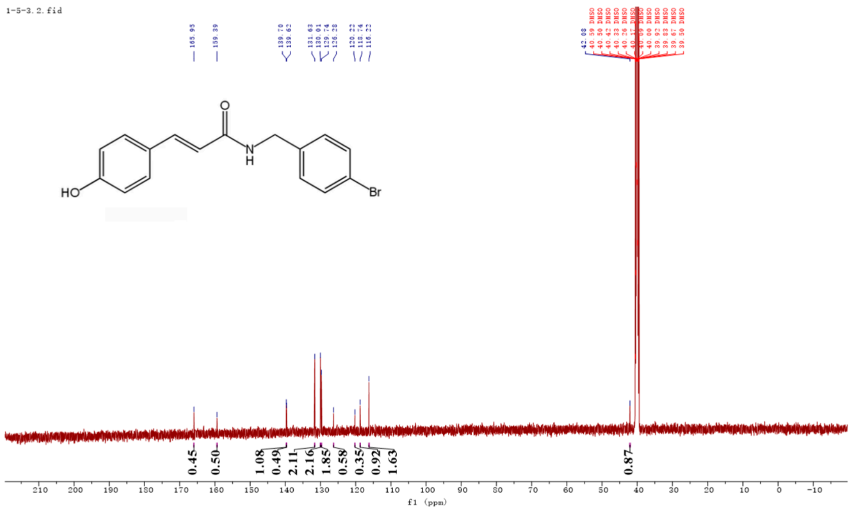

Figure S8 ^{13}C NMR spectrum of compound **BAM-HH** in DMSO.

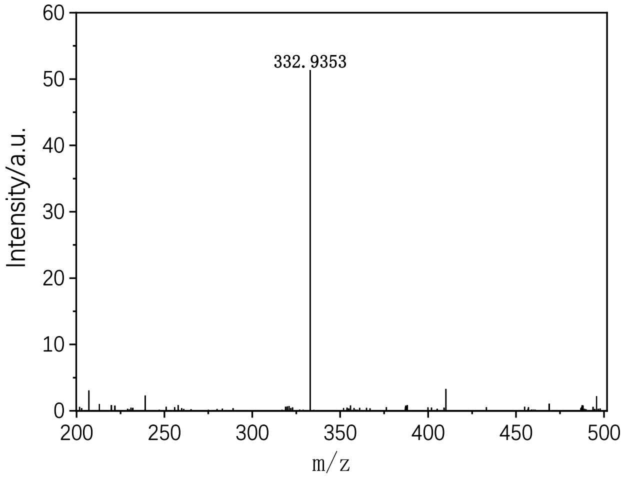

Figure S9 ESI-HRMS spectrum of compound **BAM-HH.**

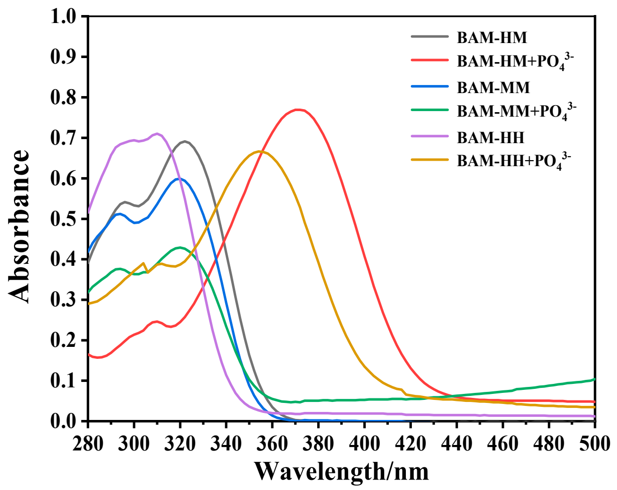

Figure S10. UV-vis titration spectra of three compounds **BAM-HM**, **BAM-MM** and **BAM-HH** in the presence of PO_4^{3-} in DMF/H_2O (pH=7.0, V/V=1/1).

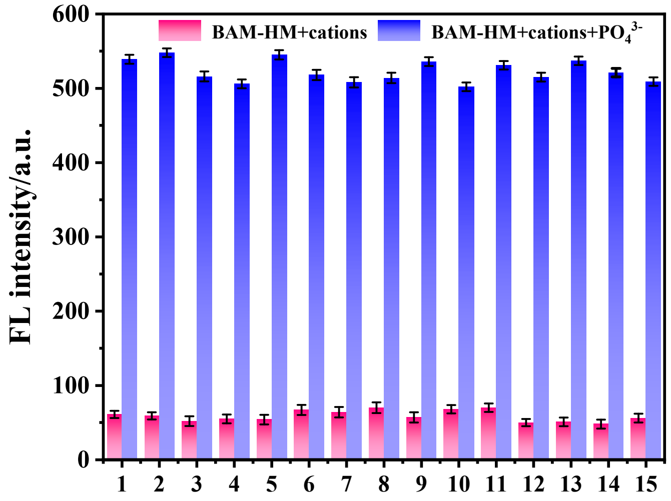

Figure S11. The fluorescence intensity of **BAM-HM** (10 μM) in the absence and presence of hydrazine coexisting with various analyties (1-15: Ca^{2+}、Cr^{3+}、Cu^{2+}、Cd^{2+}、Al^{3+}、Ni^{3+}、Co^{2+}、Ba^{2+}、Fe^{3+}、Fe^{2+}、Mg^{2+}、Zn^{2+}、Mn^{2+}、Hg^{+}、Ag^{+}).

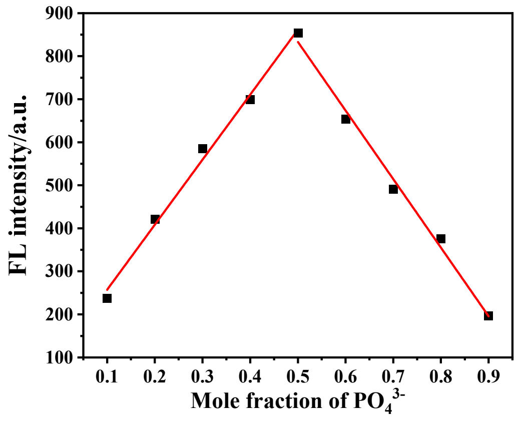

Figure S12. Job's plot for binding stoichiometry between **BAM-HM** and PO_4^{3-}.

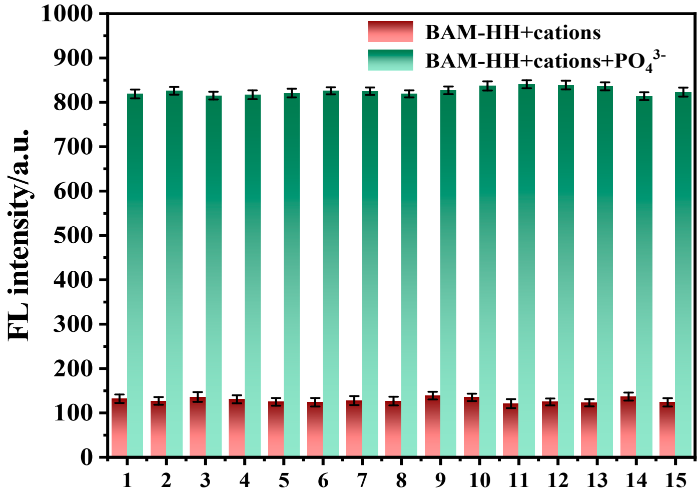

Figure S13. The fluorescence intensity of **BAM-HH** (10 μM) in the absence and presence of hydrazine coexisting with various analyties (1-15: Ca^{2+}、Cr^{3+}、Cu^{2+}、Cd^{2+}、Al^{3+}、Ni^{3+}、Co^{2+}、Ba^{2+}、Fe^{3+}、Fe^{2+}、Mg^{2+}、Zn^{2+}、Mn^{2+}、Hg^{+}、Ag^{+}).

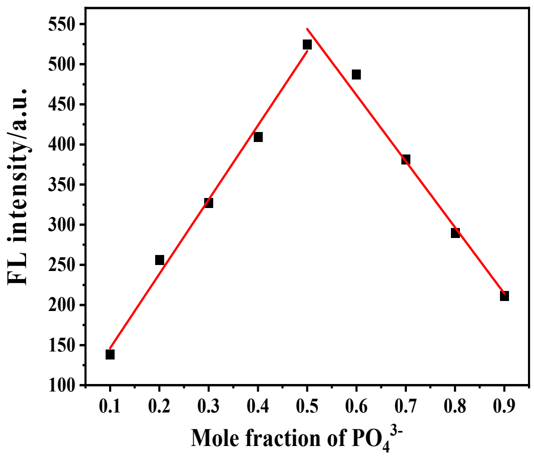

Figure S14. Job's plot for binding stoichiometry between **BAM-HH** and PO_4^{3-}

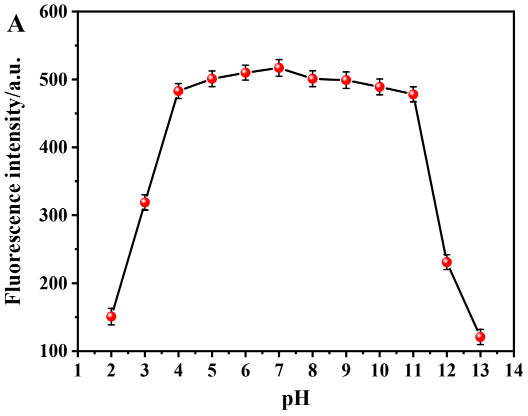

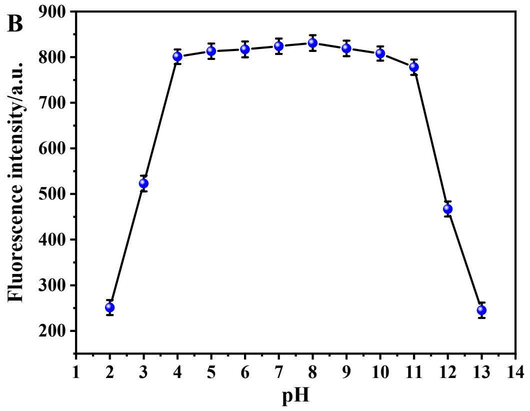

Figure S15. The effect of pH on the fluorescence intensity of **BAM-HM**- PO_4^{3-} (A) and **BAM-HH**- PO_4^{3-} (B).

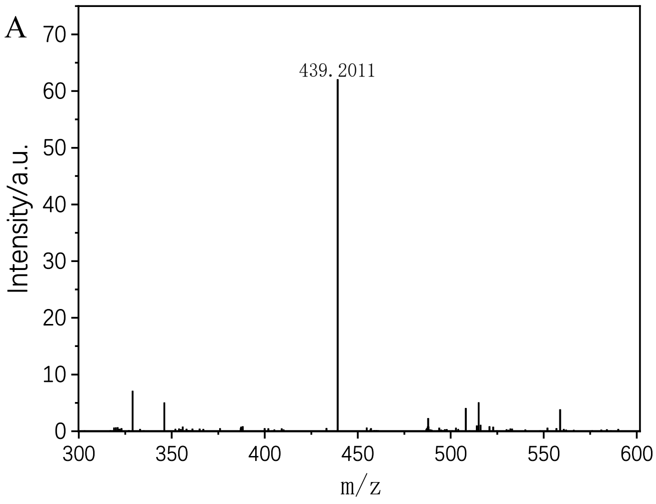

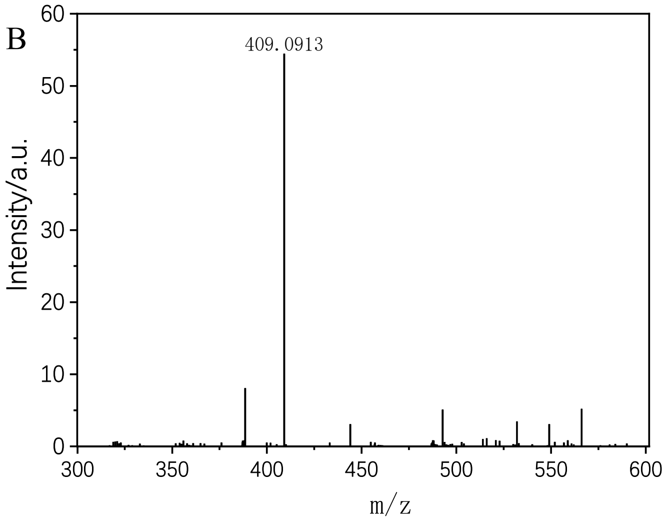

Figure S16. The HRMS spectra of **BAM-HM**+PO_4^{3-} (A) and **BAM-HH**+PO_4^{3-} (B)

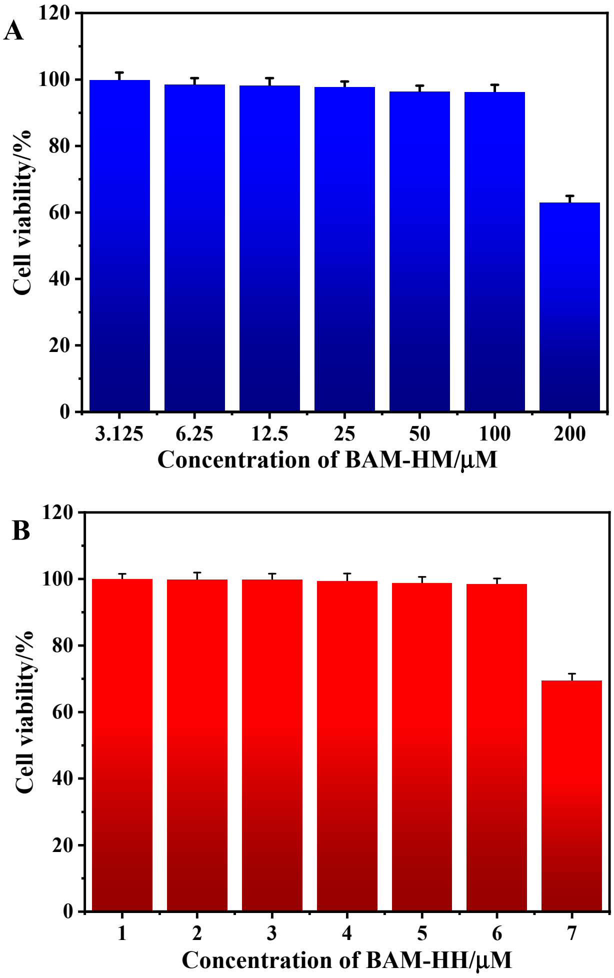

Figure S17 The survival rate HeLa cells treated with various concentrations of probe **BAM-HM** (A) and **BAM-HH** (B)

Table S1. Comparison of reported fluorescent probes for PO_4^{3-} with **BAM-HH** and **BAM-HM**

Probe	LOD	Detection medium	Reference
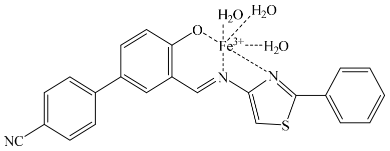	18.6 μM	EtOH/H_2O = 2:8, pH= 7.4	Spectrochim Acta A 2018 [38]
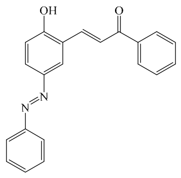	19.69 μM	DMF/H_2O=1/1	J. Mol. Struct. 2024 [39]
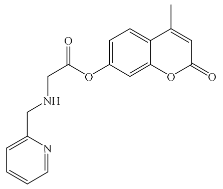	0.8 μM	100% aqueous solution	Tetrahedron 2017 [40]
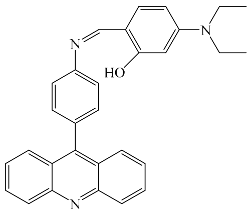	2.63 μM	methanol	Sensors and Actuators 2014 [41]
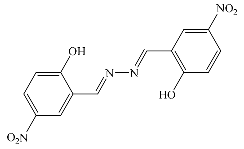	0.39 μM	10% H_2O-DMSO medium	JPP 2023 [42]
**BAM-HM**	0.612 μM	DMSO/H_2O=1/1	
**BAM-HH**	0.318 μM	DMSO/H_2O=1/1	

Table S2 Determination of PO_4^{3-} concentrations in real water samples by **BAM-HM**

Sample	Spiked /μM	Found/μM	Recovery/%
Spring water	2	1.93	96.5%
	5	5.11	102.2%
	10	10.14	101.4%
	15	15.36	102.4%
Tap water	2	2.05	102.5%
	5	4.89	97.8%
	10	9.93	99.3%
	15	15.41	102.7%

Table S3 Determination of PO_4^{3-} concentrations in real water samples by **BAM-HH**

Sample	Spiked /μM	Found/μM	Recovery/%
Spring water	2	2.09	104.5%
	5	5.13	102.6%
	10	9.78	97.8%
	15	15.45	103%
Tap water	2	1.96	98%
	5	4.91	98.2%
	10	10.49	104.9%
	15	14.78	98.5%
